# The effects of a socially evaluated cold press stressor on inhibitory gating, bradykinesia, and tremor in persons with Parkinson’s disease

**DOI:** 10.3389/fnhum.2025.1613266

**Published:** 2025-07-22

**Authors:** Andrew Zaman, Crystal Jewell, Patricia Izbicki, Elizabeth L. Stegemöller

**Affiliations:** Department of Kinesiology, Iowa State University, Ames, IA, United States

**Keywords:** Parkinson’s disease, stress, inhibitory gating, p50, paired click

## Abstract

**Introduction:**

Impaired inhibitory gating is a sensory processing symptom of Parkinson’s disease (PD) that may be associated with bradykinesia and motor inhibition. Acute stress impairs inhibitory gating in healthy adults; however, it is unclear how stress impacts inhibitory gating in people with PD.

**Methods:**

Using a Socially Evaluated Cold Pressor (SECP) to induce acute physical stress, inhibitory gating was assessed through electroencephalography (EEG) in eight individuals diagnosed with PD and 11 age- and gender-matched healthy older adults (HOAs) by measuring the p50 ratio during an auditory paired click paradigm. Kinematic measures of bradykinesia and tremor were also collected.

**Results:**

Results confirmed decreased inhibitory gating [*F*_(1,17)_ = 12.813, *p* = 0.002, *η_p_^2^* = 0.430], decreased finger tapping amplitude [*F*_(1,27)_ = 7.420, *p* = 0.011, *η_p_^2^* = 0.216], and increased postural tremor amplitude [*F*_(1,27)_ = 6.676, *p* = 0.016, *η_p_^2^* = 0.198] in both persons with PD and HOAs following the induction of an acute physical stressor, with larger differences in persons with PD. Moreover, decreases in inhibitory gating were significantly related to changes in finger tapping amplitude and postural tremor amplitude.

**Discussion:**

These findings provide evidence to suggest that stress impairs both inhibitory gating and some motor impairments in persons with PD, and that these impairments may be related. These results add to the limited literature in understanding the effects of stress on PD symptoms and may inform future potential clinical targets for therapeutics.

## Introduction

Motor impairments, such as tremor, bradykinesia, and rigidity, are hallmark symptoms of Parkinson’s disease (PD). However, in addition to motor symptoms, persons with PD also experience a number of secondary non-motor symptoms, including sensory processing impairments (Boecker et al., 1999; Gulberti et al., 2015; Kaji, 2001; Patel et al., 2014; Teo et al., 1997). One sensory processing impairment observed in persons with PD is reduced inhibitory gating (Gulberti et al., 2015; Lukhanina et al., 2009; Lukhanina et al., 2011; Teo et al., 1997), which has been associated with motor symptoms, such as bradykinesia (Lukhanina et al., 2011). Inhibitory gating is a natural pre-attentional sensory process that filters out repetitive information (Boutros and Belger, 1999; Gjini et al., 2010). Acute stress has been shown to negatively impact inhibitory gating in healthy adults (Ermutlu et al., 2005; Johnson and Adler, 1993; White and Yee, 1997). However, it is unknown how stress impacts inhibitory gating in persons with PD.

Inhibitory gating is modulated by key neurotransmitters, such as norepinephrine. For example, inhibitory gating has an inverted U-shaped relationship with norepinephrine, where both agonists and antagonists have been shown to disrupt normal gating (Adler et al., 1991; Stevens et al., 1993). Additionally, acute stressors impair inhibitory gating, proposedly by increasing the release of norepinephrine (Ermutlu et al., 2005; Johnson and Adler, 1993). Persons with PD suffer cellular loss in the locus coeruleus, a region that produces and projects norepinephrine (Vermeiren and De Deyn, 2017), and thus impaired gating in persons with PD might be mediated by low levels of norepinephrine. The basal ganglia have also been implicated in impaired inhibitory gating in PD (Gulberti et al., 2015; Lukhanina et al., 2009; Lukhanina et al., 2011; Teo et al., 1997). The basal ganglia modulates sensory information (Juri et al., 2011) and is functionally connected to prefrontal and parietal regions via subcortical loops (McHaffie et al., 2005). Furthermore, impaired gating is seen in other disorders of the basal ganglia, such as Huntington’s disease and focal dystonia (Lim et al., 2005; Teo et al., 1997; Uc et al., 2003). Involvement of the basal ganglia is also demonstrated by studies where subthalamic nucleus deep brain stimulation and ablative pallidal surgery restore normal inhibitory gating in persons with PD (Gulberti et al., 2015; Mohamed et al., 1996; Teo et al., 1998). Taken together, this evidence suggests that multiple brain areas impacted by PD are involved in inhibitory gating. Impairments in these same brain areas also underlie motor symptoms in PD. However, there is limited research that has studied how stress, inhibitory gating, and motor symptoms are related in PD.

Anecdotally, persons with PD often report that their motor symptoms worsen with stress, but research is very limited. However, animal research shows that restraint stress negatively impacts skilled reaching and grasping in a PD rat model (Smith et al., 2008). In persons with PD, gait performance worsens when they are asked to walk on a virtual elevated plank compared to a virtual plank on the ground. Although the authors interpret this as stress negatively impacting gait, no stress responses were measured (Ehgoetz Martens et al., 2015). There remains limited research on the relationship between stress and other cardinal motor symptoms, such as bradykinesia and tremor, in persons with PD. Thus, the main purpose of this study was to examine how an acute physical stressor [Socially Evaluated Cold Pressor (SECP)] impacts inhibitory gating (p50 ratio), bradykinesia, and tremor in persons with PD. In addition, this study sought to determine the relationship between inhibitory gating and motor symptoms in persons with PD. Given the aforementioned gaps in knowledge, we hypothesize that the SECP task will impair inhibitory gating in both persons with PD and healthy older adults (HOAs). We also hypothesize that the SECP task negatively impacts upper extremity bradykinesia and tremor in persons with PD but not HOAs. Finally, given that previous research has shown that reduced inhibitory gating is associated with bradykinesia in PD (Lukhanina et al., 2011), we hypothesize that there will be a negative association between inhibitory gating and bradykinesia and tremor, where decreases in inhibitory gating will be associated with an increase in symptom severity.

## Methods

### Participants

Eight participants diagnosed with idiopathic PD (mean age 67.8 ± 4.7 years; 3 males and 5 females), and 11 age- and gender-matched HOAs (mean age 68.7 ± 5.0 years) completed this study (Table 1). Participants with PD were recruited via the Iowa State University Alternative Medicine and Music for PD Lab Database, which consists of a list of individuals diagnosed with PD who have indicated interest in research opportunities. HOAs were recruited via word of mouth. The primary inclusion criterion for this group was being between the ages of 50 and 80 years (the average age range of a person with PD) (Wirdefeldt et al., 2011). Additional inclusion criteria for participants with PD included a diagnosis of PD by a neurologist. All participants were excluded if they demonstrated any cognitive impairment (Mini-Mental Status Exam < 25), had severe hearing loss, been diagnosed with a mental disorder besides anxiety or depression, had any musculoskeletal disorders or any other brain-related conditions, used tobacco, illicit drugs, or excessive amounts of alcohol, and had either a systolic blood pressure (SBP) above 140 mmHg or a diastolic blood pressure above 90 mmHg during the initial screening. One HOA was excluded during the initial blood pressure screening to avoid any potential cardiac events during the SECP task. All participants provided written informed consent. All procedures were approved by the Iowa State University Institutional Review Board (approval #: 15–499) and were performed in accordance with relevant guidelines and regulations.

**Table 1:** Demographic and questionnaire information.

Demographic/Questionnaire	PD	HOA
Age (years)	67.8 ± 4.7	68.7 ± 5.0
Gender (% male)	33.3 ± 50.0	33.3 ± 50.0
MDS – UPDRS	66.9 ± 5.8	N/A
H&Y	2.3 ± 0.1	N/A
Years diagnosed	9.9 ± 1.7	N/A
MMSE	29.3 ± 0.7	29.7 ± 0.6
GDS	6.5 ± 4.5	3.8 ± 3.7
PSS	13.5 ± 5.6	9.2 ± 6.6
STAI1	33.3 ± 8.5	28 ± 10.9
STAI2**	38.3 ± 8.7	29.1 ± 7.6

Means and standard deviations for all demographic measures and cognitive questionnaires. For between-subjects comparisons, **p < 0.01. HOA, healthy older adult; PD, Parkinson’s disease; MDS – UPDRS, Movement Disorder Society – Unified Parkinson’s Disease Rating Scale; H&Y, Hoehn and Yahr; MMSE, Mini-Mental Status Exam; MOCA, Montreal Cognitive Assessment; GDS, Geriatric Depression Scale; PSS, Perceived Stress Scale; STAI1, State and Trait Anxiety Inventory (State); STAI2, State and Trait Anxiety Inventory (Trait).

### Procedure

Participants were scheduled for two visits within a 1-week timespan. During the initial visit, participants were screened for cognitive impairment with the Mini-Mental Status Exam (MMSE) and high blood pressure using an automated Omron blood pressure cuff. Participants then provided demographic information and completed a battery of questionnaires that are reliable and valid measures for persons with PD, including the Geriatric Depression Scale (GDS) (Ertan et al., 2005) and the State and Trait Anxiety Inventory (STAI) (Yang et al., 2019). The Perceived Stress Scale (PSS) was also collected. While this scale has not been tested for its psychometric properties in persons with PD, it has been shown to be reliable and valid in older adults (Jiang et al., 2017; Lee, 2012). Participants with PD also completed the Movement Disorders Society-Unified Parkinson’s Disease Rating Scale (MDS-UPDRS) (Goetz et al., 2008). The same trained rater completed the collection and scoring of the MMSE and MDS-UPDRS across all participants and visits. Table 1 shows the means and standard deviations of demographic and questionnaire data.

During visit 1, participants were informed about the dietary and medical restrictions they were requested to follow before their second visit. These included limiting their caffeine intake to one cup (8 oz.) of a caffeinated beverage finished at least 3 h before the start of the second visit and refraining from alcoholic beverages during the 24 h before the start of the second visit. If they were non-compliant with any of these restrictions, participants were asked to reschedule. All participants were also asked to eat 1–2 h before the second visit. Participants with PD were asked to take their normal PD medication 1 h before the beginning of the second visit. The second visit was scheduled to align with their normal medication times. The second visit’s starting times were scheduled between 12 PM and 3 PM.

**Figure 1:**
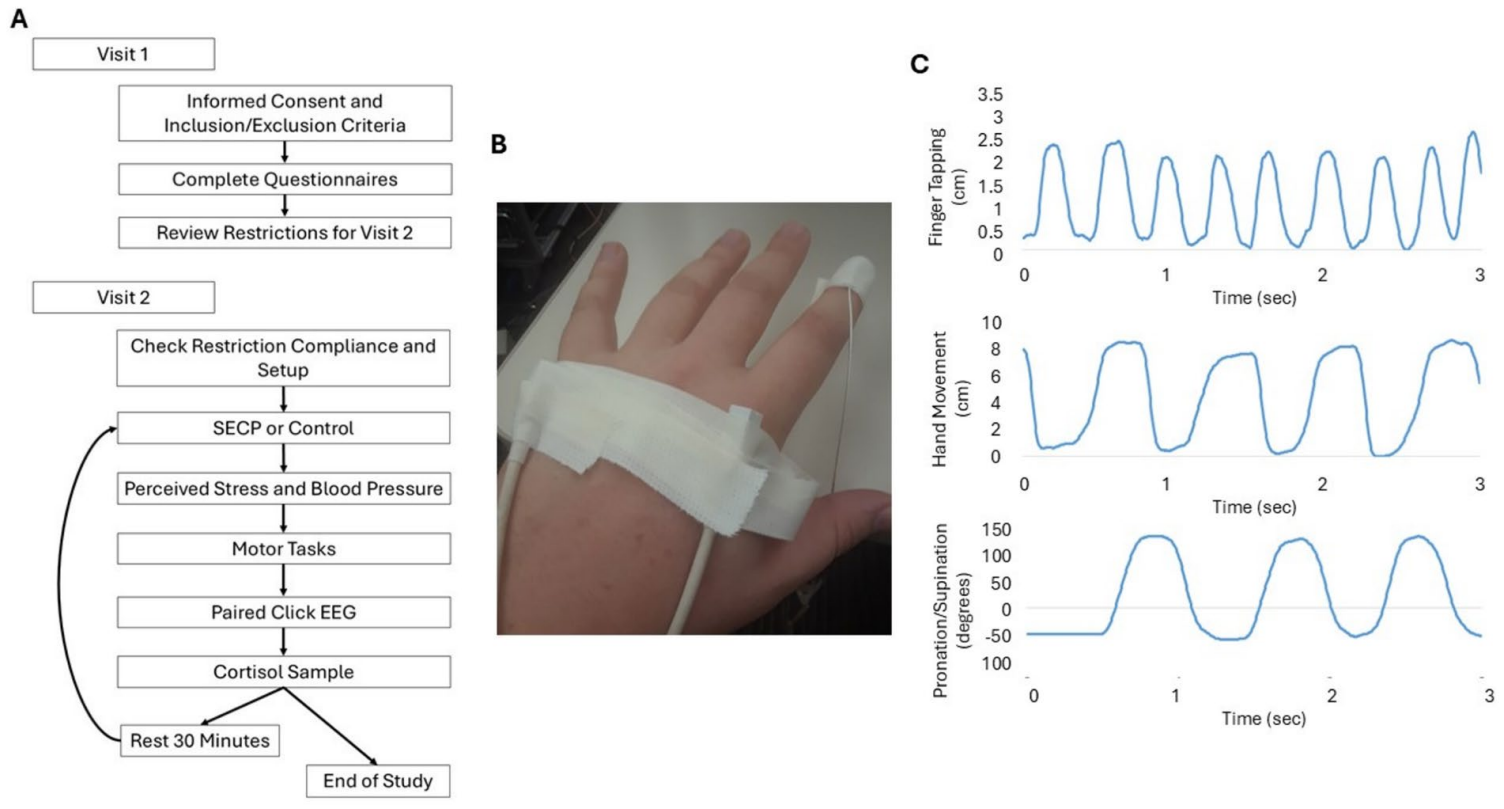
Study Design. **(A)** The flow of data collection for each visit. **(B)** Example of placement of the electrodes for motion capture. (C) Examples of raw data collection for each movement outcome from the sensors.

During the second visit, adherence to the dietary and medical restrictions and recommendations was first reviewed to ensure compliance. Then, an electroencephalogram (EEG) and electromagnetic sensors (Ascension trackSTAR, Victoria) were fitted on the participant. Electromagnetic sensors were placed on the tip of the index finger, the metacarpophalangeal joint of the index finger, and the metacarpophalangeal joint of the pinky finger on the most affected side (Figure 1B). Next, a salivary cortisol sample was collected, and then the participants completed either the physical stress intervention (SECP task) or the control intervention (warm water hand bath). The order was counterbalanced. After completing either the SECP or control task, blood pressure was obtained with an automated Omron blood pressure cuff. Then, participants verbally reported how stressful they thought the task was on a Likert scale from 1 to 10, with respective response anchors of “not stressful at all” and “most stressful thing I can imagine.” Cortisol, blood pressure, and perceived stress were measured to assess the effectiveness of the stress and control interventions. EEG data were then collected during a paired click paradigm to examine inhibitory gating. Within 5 min of completing the SECP and control tasks, participants completed the bradykinesia and tremor tests. Twenty-five minutes after completing the paired click paradigm, a second salivary cortisol sample was collected from each participant. Following this, participants rested or read a magazine or a book for half an hour. After the rest period, participants completed the same series of steps and tasks again with the uncompleted intervention (i.e., warm water bath or SECP). See Figure 1A for the order of first and second visit procedures.

### Stress and control tasks

The physical stress intervention was a SECP task in which the participants placed their hands up to the wrist in an ice water bath (2–4° C) for 90 s. Participants were also made aware that this task was being video recorded and that their facial expressions would be judged by trained experimenters at a later time. During the control condition, participants placed their hand in warm water (36–38°C) for 90 s, and participants were reminded that they were not being videotaped, and the camera was moved out of sight.

### Paired click, EEG data acquisition, and analysis

Participants completed 80 trials of the paired click paradigm to examine inhibitory gating. Inhibitory gating is typically evaluated using a paired click paradigm where pairs of simple auditory stimuli elicit the EEG p50 auditory components (Lijffijt et al., 2009). During the paired-click paradigm, the first click is referred to as S1 and is followed shortly thereafter by the second click, referred to as S2. The most common inhibitory gating measure is the S2/S1 ratio of the p50 component (p50 ratio) (Boutros and Belger, 1999; Guterman et al., 1992; Jones et al., 2016). The normal gating response is indicated by a low S2/S1 ratio, with a large amplitude response to S1 and a reduced amplitude response to S2.

During this task, the participants were sitting comfortably in a semi-reclined armchair in a quiet, well-lit room. Participants were instructed to keep their movements to a minimum and their eyes open and focused on a piece of tape stuck to a desk 5 feet in front of them. Participants were also instructed to refrain from falling asleep.

To collect the potentials, a 64-electrode EEG cap was fitted according to the international 10–20 system (Biosemi, Amsterdam, Netherlands). Eye movements were monitored with electrodes placed above and below the right eye for filtering purposes. A single common reference electrode was placed over the mastoid process ipsilateral to the participant’s most affected side (HOAs were matched), and the ground electrode was placed on the forehead just above the nose and between the eyebrows. The electrode of interest was Cz, which is located in the midline at the ‘vertex’ or top of the head. Impedances were checked to make sure they are below 5 kohms before beginning the data collection. Signals were recorded at 2 kHz and amplified. During the task, auditory clicks were presented through speakers at an 80 dB hearing level. The auditory stimuli were pairs of identical auditory clicks (1,000-Hz tone) with a duration of 20 ms and an interval of 500 ms between clicks. Eighty trials were presented with a 7-s interval between trials. The time for each recording epoch was 300 ms before the first pair of clicks until 1 s following the second click. The auditory tones were collected simultaneously by the EEG data collection system (The Motion Monitor, Innsport, Chicago) to ensure synchronization. Overall, these are the recommended methods for collecting the p50 response (Dalecki et al., 2011).

The EEG data were processed using standard methods from the Krigolson Laboratory^1^. Signals were re-referenced offline using a bipolar montage (Cz-Iz), baseline corrected, and filtered using a 0.5–45 Hz 4th-order dual-pass Butterworth band-pass and a 60-Hz notch filter. Next, the data were divided into smaller epochs (−200 ms to 300 ms) around each auditory tone. Finally, independent component analysis followed by inspection of the data was used to identify artifacts, any trials with voltage on any channel exceeding a 10 μV/ms gradient, and an absolute voltage difference >100 μV. These trials were eliminated, resulting in 65–70 epochs per participant entered for analysis.

The P50 ratio (S2/S1) was the measure of habituation (Dalecki et al., 2011). S1 (peak-to-peak method) was the difference in amplitudes in the positive deflection between 35 and 65 ms and the most negative deflection between 15 and 45 ms of the first stimulus. S2 was the difference of those amplitudes for the second stimulus. In addition, latency measures were also calculated for S2 and S1, where the latency value reflected the timing difference between the maximum positive deflection and the auditory stimulus. A clear p50 component was interpretable in both the S1 and S2 in both conditions (SECP and control) for 19 participants (8 PD and 11 HOAs).

### MDS-UPDRS motor data acquisition and analysis

Participants were seated comfortably in a semi-reclined armchair in a quiet, well-lit room. Select MDS-UPDRS motor tests for bradykinesia (sections 3.4 finger tapping, 3.5 hand movements, 3.6 pronation-supination movements of hands) and tremor (3.15 postural tremor of the hands, and 3.17 rest tremor of the hands) were examined in the same order for all participants. Only seated tests were chosen so that data could be captured in a timely manner after the SECP condition. In addition, rigidity was not collected as the lab was not equipped with the methodology to measure rigidity. Standard instructions for these motor tests were provided, with the modification that participants were instructed to complete each of the tests for 10 s to allow for the collection of kinematic data. For participants with PD, the least affected side was placed in the water (SECP or warm water control), so data were only recorded on the most affected side. For HOAs, they were matched to participants with PD and used the same counterpart for data collection.

**Figure 2:**
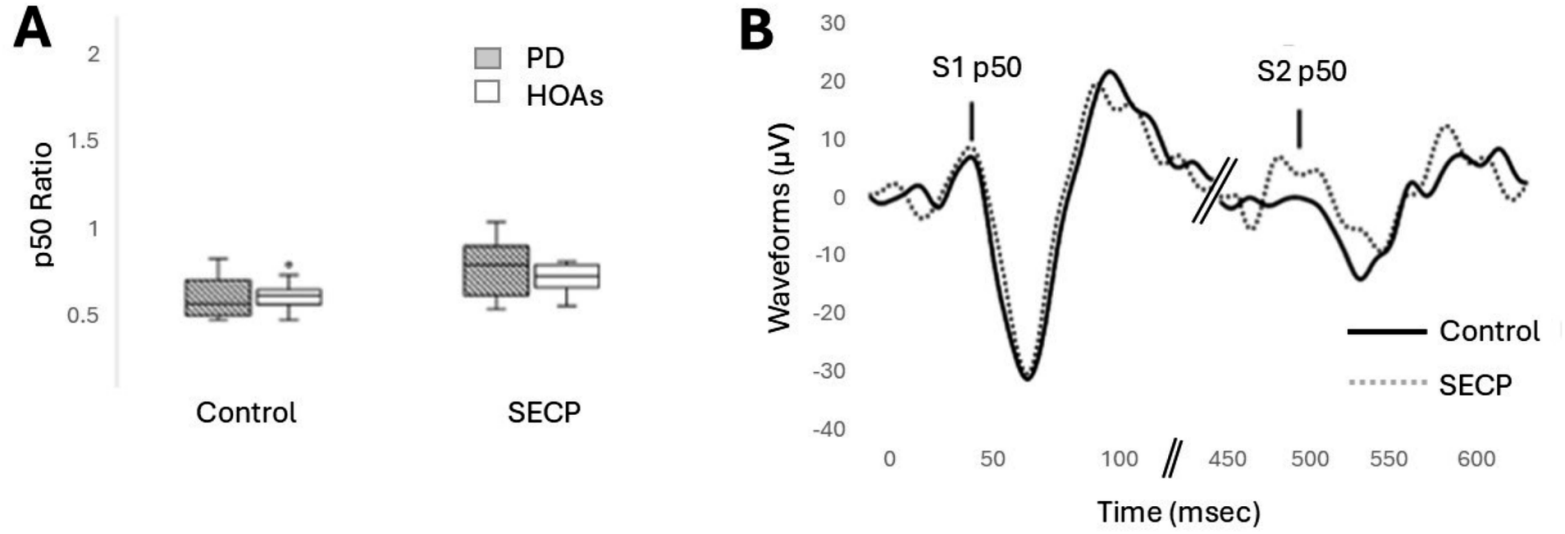
The effects of the SECP and Control Tasks on the p50 response. **(A)** p50 ratio for both groups and both conditions. **(B)** Example EEG waveform, zoomed in, from one person with PD in both conditions.

For all movement tests, the primary outcome measures were movement rate and amplitude. Movement rate was measured in cycles per second (Hz), with the end of each cycle marked by: (1) when the index finger reached a minimum on the *y*-axis (i.e., touched the thumb for the finger tapping task or palm of the hand) for finger tapping and hand movements (Figure 1C) or (2) reached the maximum rotation when pointing the palm to the floor (pronation-supination of the hand) (Figure 1C). For finger tapping and hand movements, amplitude was analyzed by examining the magnitude of movement of the index finger in relation to the metacarpophalangeal joint of the index finger. For pronation-supination movements of hands, the amplitude of the movement was analyzed by examining the degree of rotation between the metacarpophalangeal joints of the index and pinky fingers. For the tests of postural and resting tremor, the amplitude of tremor was measured by examining the magnitude of tremor frequency movements (3–8 Hz) using the tip of the index finger. The largest tremor seen during the tremor recording periods was recorded as the maximum amplitude. To measure interruptions, decrements in amplitude, and slowing, variability of movement was examined by calculating the coefficient of variation (CV; standard deviation/mean) for each of the outcome measures with the exception of max amplitude.

### Cortisol analysis

On the day of collection, salivary cortisol samples were stored in a −20°C freezer within 30 min. Cortisol was analyzed with the Salimetrics^®^ Cortisol Enzyme Immunoassay Kit (RRID AB_2801306). The kit uses a competitive immunoassay in which cortisol competes with cortisol conjugated to horseradish peroxidase for the antibody binding sites. To determine the cortisol reactivity, the area under the curve was calculated using the baseline and the 25-min post intervention samples for each condition.

### Statistical analysis

An independent samples *t*-test was used to examine any group differences on demographic and questionnaire outcome measures. A 2 condition (SECP, control) × 2 group (PD, HOA) repeated measures ANOVA was completed for measures of stress (perceived stress, blood pressure, and cortisol) to confirm that the SECP task initiated an increase in stress for participants. To test the hypothesis that the SECP task impaired inhibitory gating (higher p50 ratios) in both persons with PD and HOAs, a 2 condition (SECP, control) × 2 group (PD, HOA) repeated measures ANOVA was used to determine differences in inhibitory gating (p50 ratio). To test the hypothesis that the SECP task would negatively impact bradykinesia and tremor in persons with PD but not in HOAs, a group (PD, HOA) × 2 (SECP, control) repeated measures ANOVA was used to determine differences in each tremor (amplitude, amplitude CV, and max amplitude) and upper extremity bradykinesia (movement rate, movement rate CV, amplitude, and amplitude CV) kinematic outcome measure. For all repeated measures ANOVAs, partial eta squared (*η_p_^2^*) effect sizes were calculated. Significance was set at *α* = 0.05. For all *post hoc* analyses when appropriate, a Bonferroni correction was used to interpret statistical significance.

A repeated measures Pearson’s correlation (Bakdash and Marusich, 2017) was used to examine the relationship between the p50 ratio and bradykinesia and tremor during the control and stress conditions. Repeated measures correlations were used to examine within-individual associations for measures assessed on two or more occasions for multiple individuals. Repeated measures correlations do not violate the assumption of independence of observations, and estimate the association shared among individuals. Repeated measures correlation uses an analysis of covariance (ANCOVA) to adjust for inter-individual variability by removing between-participants variance and provides the best linear fit for each participant using a parallel regression line with varying intercepts. Similar to the Pearson correlation coefficient (*r*), repeated measures correlation is bounded by −1 to 1 and represents the strength of the linear association between two variables. Significance was set at *α* = 0.05.

## Results

### Participants

Although participants with PD have higher scores than HOAs on the mood questionnaires, only the STAI2 reached significance. Participants with PD had higher trait anxiety than the HOAs [*t*_(28)_ = 3.086, *p* = 0.005, Mean Difference (*MD*) = +9.2, *d* = 1.126]. No demographic variables were found to be significantly different (Table 1).

### Stress outcome measures

For perceived stress, both participants with PD and HOAs similarly rated the SECP condition more stressful than the control condition. Results revealed a main effect of condition [*F*_(1,28)_ = 147.393, *p* < 0.001, *η_p_^2^* = 0.840]; the SECP task was rated higher than the control condition (*MD* = +5.2, *d* = 2.256). There was no main effect of group [*F*_(1,28)_ = 0.267, *p* = 0.609, *η_p_^2^* = 0.009], or an interaction effect for group x condition [*F*_(1,28)_ = 0.006, *p* = 0.938, *η_p_^2^* = 0.000].

Blood pressure was elevated similarly for participants with PD and HOAs after the SECP condition compared to the control condition. There was a main effect of condition for both systolic [*F*_(1,28)_ = 8.003, *p* = 0.009, *η_p_^2^* = 0.222] and diastolic blood pressure [*F*_(1,28)_ = 5.543, *p* = 0.026, *η_p_^2^* = 0.165]. The participants had higher systolic blood pressure (*MD* = +6.4 mmHg, *d* = 0.518) and diastolic blood pressure (*MD* = +2.8 mmHg, *d* = 0.430) after the SECP is compared to the control condition. There was no main effect of group for systolic blood pressure [*F*_(1,28)_ = 2.138, *p* = 0.155, *η_p_^2^* = 0.071] or diastolic blood pressure [*F*_(1,28)_ = 0.360, *p* = 0.553, *η_p_^2^* = 0.013]. There were no interaction effects of group x condition for systolic blood pressure [*F*_(1,28)_ = 0.889, *p* = 0.354; *η_p_^2^* = 0.031] or diastolic blood pressure [*F*_(1,28)_ = 0.767, *p* = 0.388; *η_p_^2^* = 0.027].

Similar to the other stress outcome measures, cortisol was similarly raised in both participants with PD and HOAs after the SECP condition was compared to the control condition. There was a main effect of condition [*F*_(1,28)_ = 6.780, *p* = 0.015 *η_p_^2^* = 0.195]; cortisol area under the curve (AUC) values were higher after the SECP was compared to the control condition (*MD* = +13.4 ng/dL/h, *d* = 0.479). There was no main effect of group [*F*_(1,28)_ = 0.187, *p* = 0.699, *η_p_^2^* = 0.007] and no interaction effects for group x condition [*F*_(1,28)_ = 0.596, *p* = 0.447, *η_p_^2^* = 0.021].

### Inhibitory gating

No differences in inhibitory gating between the participants with PD and HOA were revealed, but inhibitory gating was reduced in both participants with PD and HOA after the SECP condition was compared to the control condition. Figure 2A shows the results for inhibitory gating, and Figure 2B shows an example of the EEG waveform from one person with PD in both conditions. Results revealed a main effect of condition [*F*_(1,17)_ = 12.813, *p* = 0.002, *η_p_^2^* = 0.430]. In general, there was less inhibitory gating following the SECP condition compared to the control condition (MD = +0.20, *d* = 0.760). No main effect of group [*F*_(1,17)_ = 0.736, *p* = 0.403, *η_p_^2^* = 0.042] or interaction effects for condition x group [*F*_(1,17)_ = 1.971, *p* = 0.178, *η_p_^2^* = 0.104] were found.

### Bradykinesia and tremor

**Figure 3:**
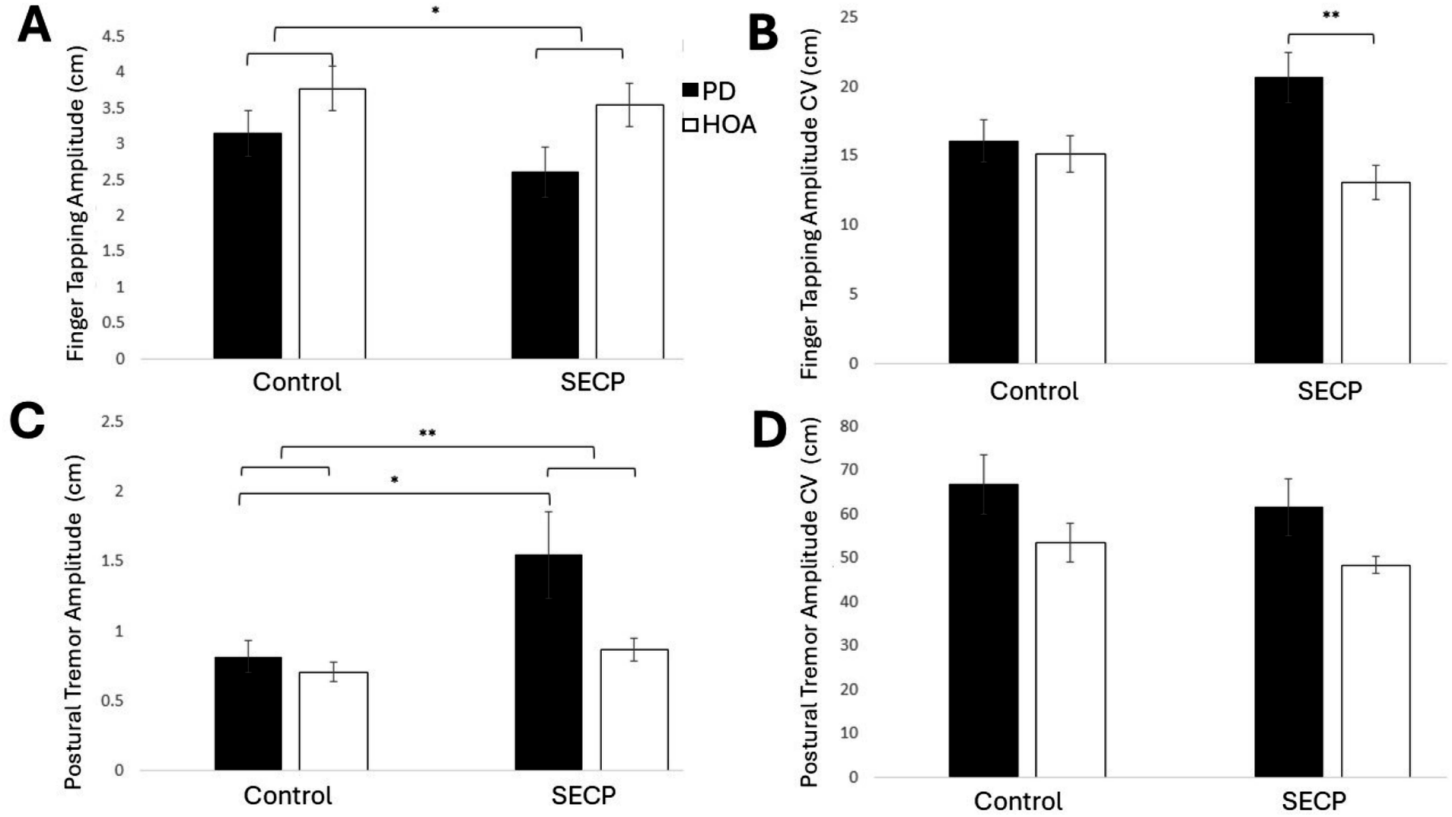
The effects of the SECP and Control Tasks on finger tapping and postural tremor. **(A)** Finger tapping amplitude for both groups and both conditions. **(B)** Finger tapping amplitude CV for both groups and both conditions. **(C)** Postural tremor amplitude for both groups and both conditions. **(D)** Postural tremor amplitude CV for both groups and both conditions. Standard error bars are shown. Small horizontal bars show between-group differences. Long horizontal bars show within-group differences. Long horizontal bars connected with short horizontal bars show the main effect of the condition. *p < 0.05, **p < 0.01.

Overall, for finger tapping, no differences in movement rate or movement amplitude were revealed between participants with PD and HOAs, but amplitude variability (i.e., CV) was significantly greater in participants with PD than those with HOAs. After the SECP, amplitude CV increased in persons with PD and decreased in HOAs. Additionally, amplitude significantly decreased in both groups after the SECP. Figure 3A shows the results for finger tapping amplitude, and Figure 3B shows the results for finger tapping amplitude CV. A main effect of condition on finger tapping amplitude [*F*_(1,27)_ = 7.420, *p* = 0.011, *η_p_^2^* = 0.216] was revealed. Participants had smaller finger taps following the SECP condition (*MD* = −0.4 cm, *d* = 0.496). No main effect of condition was found for movement rate [*F*_(1,27)_ = 2.006, *p* = 0.168, *η_p_^2^* = 0.069], amplitude CV [*F*_(1,27)_ = 0.815, *p* = 0.375, *η_p_^2^* = 0.029], or movement rate CV [*F*_(1,27)_ = 0.495, *p* = 0.488, *η_p_^2^* = 0.018]. A main effect of group on amplitude CV [*F*_(1,27)_ = 7.552, *p* = 0.011, *η_p_^2^* = 0.219] was revealed. The variability in amplitude was greater in persons with PD (*MD* = +3.4, *d* = 0.739). No main effect of group was found for movement amplitude [*F*_(1,27)_ = 3.238, *p* = 0.083, *η_p_^2^* = 0.107], movement rate [*F*_(1,27)_ = 0.402, *p* = 0.531, *η_p_^2^* = 0.015], or movement rate CV [*F*_(1,27)_ = 1.147, *p* = 0.294, *η_p_^2^* = 0.041]. The results revealed a condition x group interaction for amplitude CV [*F*_(1,27)_ = 5.501, *p* = 0.027, *η_p_^2^* = 0.169] where the finger tapping amplitude CV increased in persons with PD (*MD* = +4.6, *d* = 0.519) and decreased in HOAs (*MD* = −2.0, *d* = 0.326), following the SECP condition. *Post hoc* analysis found that persons with PD had a significantly higher amplitude CV than HOAs during the SECP condition [*t*_(27)_ = 3.516, *p* = 0.002, *MD* = 7.6, *d* = 1.305], but amplitude CV was similar between the two groups during the control condition [*t*_(27)_ = 0.386, *p* = 0.702, *MD* = 0.8, *d* = 0.175]. No condition x group interaction effects were found for amplitude [*F*_(1,27)_ = 1.161, *p* = 0.291, *η_p_^2^* = 0.041], movement rate [*F*_(1,27)_ = 0.056, *p* = 0.815, *η_p_^2^* = 0.002], or movement rate CV [*F*_(1,27)_ = 0.000, *p* = 0.989, *η_p_^2^* = 0.000].

For both repetitive hand movements, there were no differences between conditions or groups. For repetitive hand movements, no main effects of condition [*F*_(1,28)_ > 0.059, *p* < 0.809, *η_p_^2^* < 0.083], group [*F*_(1,28)_ < 3.242, *p* > 0.083, *η_p_^2^* < 0.104], or condition × group interaction effect [*F*_(1,28)_ < 1.195, *p* > 0.284, *η_p_^2^* < 0.041] were revealed for any outcome measure.

For pronation-supination of the hand, participants with PD were slower and smaller than HOAs, but the effect of the SECP condition was similar between groups. There were no main effects of condition on any of the pronation-supination of the hand outcome measures [*F*_(1,27)_ < 2.820, *p* > 0.105, *η_p_^2^* < 0.095]. However, a main effect of group for amplitude [*F*_(1,27)_ = 7.148, *p* = 0.013, *η_p_^2^* = 0.209] and movement rate [*F*_(1,27)_ = 9.086, *p* = 0.006; *η_p_^2^* = 0.252] was revealed, where persons with PD had less pronation-supination of the hand rotation (*MD* = −24.4⁰, *d* = 0.926), and slower rotations (*MD* = −0.4 Hz, *d* = 1.001) than HOAs. No main effect of group was found for amplitude CV [*F*_(1,27)_ = 0.073, *p* = 0.788, *η_p_^2^* = 0.003], or movement rate CV [*F*_(1,27)_ = 0.047, *p* = 0.829, *η_p_^2^* = 0.002]. The results revealed no condition x group interaction effects [*F*_(1,27)_ < 1.535, *p* > 0.226; *η_p_^2^* > 0.054].

Participants with PD had more postural tremor than HOAs. Postural tremor increased in both groups after the SECP condition compared to the control condition. Figure 3C shows postural tremor amplitude, and Figure 3D shows postural tremor amplitude CV. There was a main effect of condition on postural tremor amplitude [*F*_(1,27)_ = 6.676, *p* = 0.016, *η_p_^2^* = 0.198], where amplitude was greater following the SECP condition (*MD* = 0.42 mm, *d* = 0.473). There was no effect of condition for any other measures [*F*_(1,27)_ < 3.064, *p* > 0.091, *η_p_^2^* < 0.102]. There was a main effect of group on max amplitude [*F*_(1,27)_ = 8.727, *p* = 0.006, *η_p_^2^* = 0.244] and amplitude CV [*F*_(1,27)_ = 5.666, *p* = 0.025, *η_p_^2^* = 0.173], but not for amplitude [*F*_(1,27)_ = 3.302, *p* = 0.080, *η_p_^2^* = 0.109]. In general, persons with PD had a greater amplitude (*MD* = 0.36 mm, *d* = 1.48), max amplitude (*MD* = 1.88 mm, *d* = 0.813), and amplitude CV (*MD* = 13.8, *d* = 0.677) than HOAs. No condition x group interaction effects were found [*F*_(1,27)_ < 2.494, *p* > 0.126, *η_p_^2^* < 0.085].

Participants with PD also had more resting tremor than HOAs, but there was no effect of the SECP condition in either group. No main effect of condition for any resting tremor measures was revealed [*F*_(1,27)_ < 2.412, *p* > 0.132, *η_p_^2^* < 0.079]. A main effect of group on amplitude [*F*_(1,27)_ = 5.440, *p* = 0.027, *η_p_^2^* = 0.163], and max amplitude [*F*_(1,27)_ = 6.012, *p* = 0.021, *η_p_^2^* = 0.077] was revealed. Persons with PD had a higher amplitude resting tremor (*MD* = 0.62 cm, *d* = 0.749) and a higher max amplitude resting tremor (*MD* = 2.32 cm, *d* = 0.862) than HOAs. There was no main effect of group on amplitude CV [*F*_(1,27)_ = 2.349, *p* = 0.137, *η_p_^2^* = 0.077]. No condition x group interaction effects were found [*F*_(1,27)_ < 0.915, *p* > 0.347, *η_p_^2^* < 0.032] (Table 2).

**Table 2:** Means and standard error for all outcome measures for both groups and both conditions.

	PD control	HOA control	PD SECP	HOA SECP
Stress measures				
Perceived Stress^aaa^	1.20 ± 0.56	1.00 ± 0.00	6.40 ± 2.53	6.13 ± 2.27
SBP (mmHg)^aa^	121.3 ± 13.0	130.5 ± 16.2	129.7 ± 15.4	134.7 ± 13.8
DBP (mmHg)^a^	75.6 ± 9.3	74.9 ± 8.0	79.5 ± 8.7	76.7 ± 8.3
Cortisol (ng/dl/h)^a^	66.7 ± 26.4	58.8 ± 23.2	76.1 ± 27.0	76.3 ± 34.2
p50 measures				
p50 Ratio^aa^	0.61 ± 0.28	0.60 ± 0.12	0.90 ± 0.47	0.72 ± 0.16
S1Amplitude (μV)	10.42 ± 5.73	6.04 ± 2.86	9.82 ± 7.56	6.01 ± 3.00
S2 Amplitude (μV)	6.31 ± 4.27	3.71 ± 2.16	7.59 ± 5.50	4.14 ± 2.00
Movement measures				
Finger Amp (cm)^a^	3.15 ± 0.32	3.77 ± 0.32	2.61 ± 0.35	3.54 ± 0.31
Finger Amp CV^b^	16.08 ± 1.55**	15.12 ± 1.34	20.65 ± 1.81**	13.09 ± 1.22
Finger MR (Hz)	3.25 ± 0.20	3.11 ± 0.14	3.42 ± 0.27	3.24 ± 0.15
Finger MR CV	12.72 ± 1.73	10.95 ± 0.99	13.27 ± 1.08	11.51 ± 1.23
Hand Amp (cm)	6.36 ± 0.57	7.50 ± 0.43	6.45 ± 0.68	7.06 ± 0.49
Hand Amp CV	14.87 ± 1.70	10.23 ± 1.93	14.59 ± 2.58	9.92 ± 1.76
Hand MR (Hz)	2.20 ± 0.17	2.24 ± 0.13	2.12 ± 0.16	2.29 ± 0.16
Hand MR CV	16.42 ± 1.74	14.14 ± 1.96	14.11 ± 2.25	11.38 ± 1.67
Pro-Sup Amp (deg)^b^	155.4 ± 6.3	184.5 ± 5.6	153.7 ± 8.3	173.5 ± 7.8
Pro-Sup Amp CV	8.15 ± 1.17	7.11 ± 1.03	7.08 ± 0.99	7.48 ± 1.00
Pro-Sup MR (Hz)^b^	1.13 ± 0.07	1.44 ± 0.08	1.18 ± 0.06	1.52 ± 0.13
Pro-Sup MR CV	11.31 ± 1.95	9.58 ± 1.73	10.86 ± 1.50	11.70 ± 1.92
Postural Tremor (mm)^a^	0.82 ± 0.11	0.71 ± 0.07	1.49 ± 0.32	0.87 ± 0.08
Postural Tremor Max (mm)^b^	2.97 ± 0.56	1.77 ± 0.21	4.62 ± 0.97	2.06 ± 0.20
Postural Tremor CV^b^	66.7 ± 6.7	53.5 ± 4.4	62.8 ± 6.6	48.4 ± 2.0
Rest Tremor (mm)^b^	0.80 ± 0.32	0.18 ± 0.02	0.82 ± 0.30	0.19 ± 0.02
Rest Tremor Max (mm)^b^	3.04 ± 1.13	0.42 ± 0.09	2.43 ± 0.84	0.40 ± 0.06
Rest Tremor CV	69.16 ± 12.64	49.21 ± 11.21	54.88 ± 6.54	41.15 ± 4.10

For a main effect of condition, ^a^*p* < 0.05, ^aa^*p* < 0.01, ^aaa^*p* < 0.001; for a main effect of group, ^b^*p* < 0.05. For *post hoc* tests within-subject effects of condition, **p < 0.01. DBP, diastolic blood pressure; SBP, systolic blood pressure; S1, stimulus 1; S2, stimulus 2; Amp, amplitude; Finger, finger tapping; MR, movement rate; Hand, hand movements; HOA, healthy older adult; PD, persons with Parkinson’s disease; Pro-Sup, pronation-supination of the hand.

### Associations between inhibitory gating, bradykinesia, and tremor

**Figure 4:**
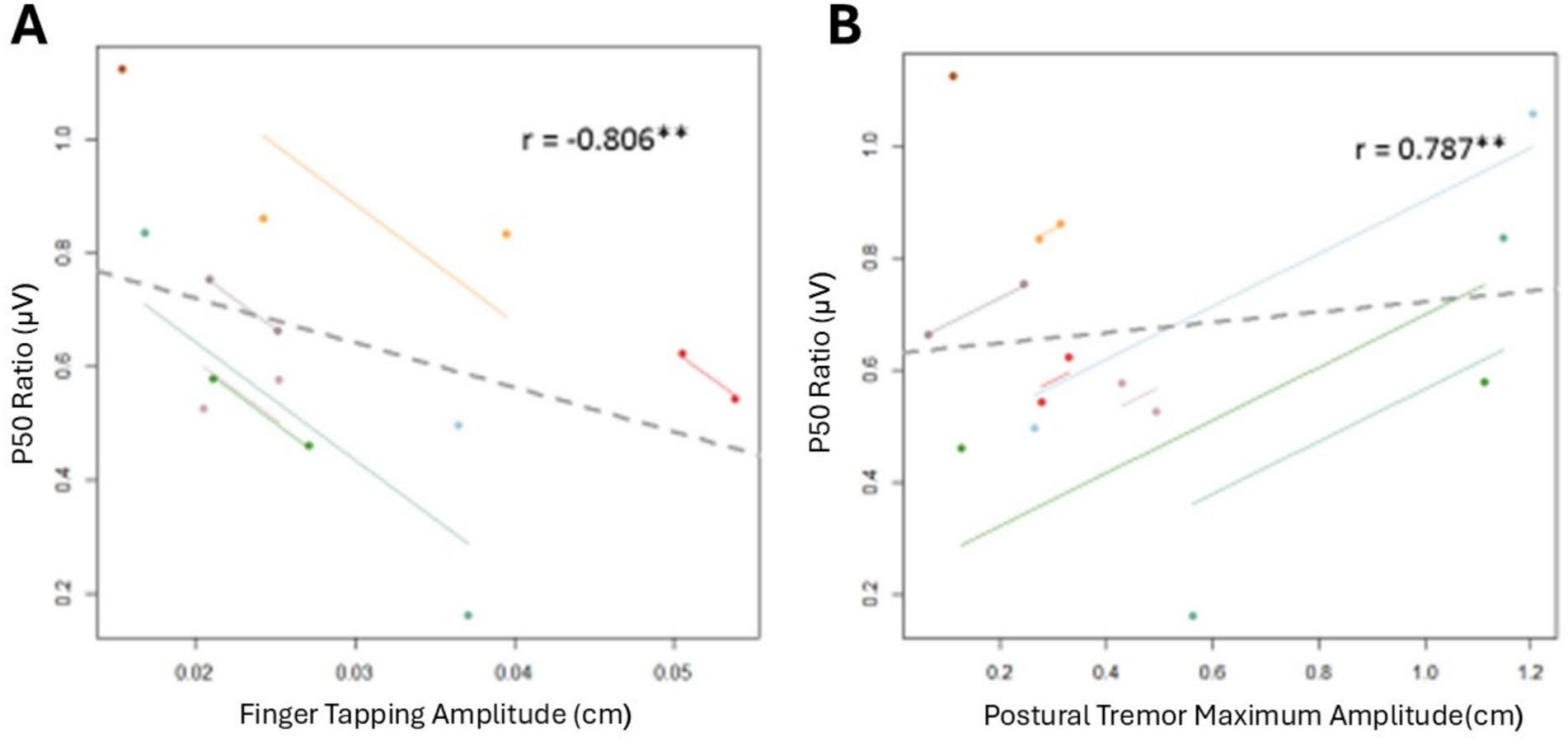
Repeated measures correlations between inhibitory gating and PD motor symptoms. Each dot represents one of two separate observations for a participant. Observations from the same participant are given the same color, with corresponding lines to show the repeated measures correlation fit for each participant. Solid color lines represent intra-individual correlations. The dotted gray line represents the fit of simple between-subject correlations. **(A)** Repeated measures correlation between p50 ratio and finger tapping amplitude. **(B)** Repeated measures correlation between p50 ratio and maximum postural tremor amplitude. **p < 0.01.

Table 3 shows the repeated measures Pearson’s *r* correlations between all movement outcome measures and the p50 ratio. Figure 4 shows the significant repeated measures correlations. Significant associations were revealed between p50 inhibitory gating and finger tapping amplitude (*r* = −0.806, *p* = 0.029) and between p50 inhibitory gating and postural tremor maximum amplitude (*r* = 0.787, *p* = 0.020). In general, as the p50 ratio increased, the finger tapping amplitude decreased, and the maximum postural tremor amplitude increased. No other correlations were significant.

**Table 3:** Pearson’s r for correlations between p50 ratio and all movement measures.

Movement amplitude and p50 ratio		Movement rate and p50 ratio		Tremor and P50 ratio	
Finger Amp (*n* = 6)	−0.806** p = 0.029	Finger Rate (*n* = 6)	−0.481 *p* = 0.274	Rest Tremor Amp (*n* = 6)	0.018 *p* = 0.969
Hand Amp (*n* = 7)	0.411 *p* = 0.312	Hand Rate (*n* = 7)	−0.462 *p* = 0.249	Rest Tremor Max (*n* = 6)	0.665 *p* = 0.072
Pro-Sup Amp (*n* = 7)	−0.412 *p* = 0.311	Pro-Sup Rate (*n* = 7)	0.052 *p* = 0.902	Rest Tremor CV (*n* = 6)	−0.470 *p* = 0.287
Finger Amp CV (*n* = 6)	0.346 *p* = 0.448	Finger Rate CV (*n* = 6)	0.313 *p* = 0.495	Postural Tremor Amp (*n* = 6)	0.481 *p* = 0.275
Hand Amp CV (*n* = 7)	0.403 *p* = 0.322	Hand Rate CV (*n* = 7)	0.114 *p* = 0.788	Postural Tremor Max (*n* = 7)	0.787** p = 0.020
Pro-Sup CV (*n* = 7)	0.126 *p* = 0.767	Pro-Sup Rate CV (*n* = 7)	0.091 *p* = 0.831	Postural Tremor CV (*n* = 7)	−0.188 *p* = 0.655

**p < 0.05. Finger, finger tapping; Hand, hand movement; Pro-Sup, pronation-supination of the hand; Amp, amplitude; Max, maximum amplitude; CV, coefficient of variation.

## Discussion

This present study aimed to test the effects of a physical stressor, SECP task, on inhibitory gating, bradykinesia, and tremor in persons with PD. The results revealed that the SECP task was successful in increasing stress-related measures (perceived stress, blood pressure, and cortisol) and decreasing inhibitory gating in both HOAs and persons with PD. The SECP task decreased inhibitory gating to a greater degree in persons with PD (*MD* = +0.293) compared to HOAs (*MD* = +0.127), but did not reach significance. For bradykinesia and tremor, the SECP task only significantly impacted finger tapping amplitude and postural tremor amplitude. Results revealed that the SECP task reduced finger tapping amplitude in both persons with PD and HOAs. However, variability in finger tapping amplitude increased in persons with PD while variability decreased in HOAs after the SECP task. Similarly, postural tremor amplitude increased following the SECP task, with a greater, though not significant, increase in postural tremor amplitude in persons with PD compared to HOAs. Interestingly, the SECP task did not affect movement rate in any of the movement tests. Finally, results revealed that decreased inhibitory gating was associated with smaller finger tapping amplitude and increased postural tremor maximum amplitude. Taken together, these results suggest that inhibitory gating may be involved in the negative effect of physical stress on some PD motor symptoms.

### Inhibitory gating

During the control condition, persons with PD and HOAs had similar inhibitory gating ratios, and stress decreased inhibitory gating in both groups. While not statistically significant, there was a medium to large condition by group interaction effect size (*η_p_^2^* = 0.104), where the SECP task resulted in a decrease in inhibitory gating in persons with PD that was more than double that observed in the HOAs. This suggests that in persons with PD, stress may impair inhibitory gating to a greater degree. While a larger sample size is warranted, the lack of significant difference in this study may also be explained by the stage of PD progression of the participant sample. The sample of persons with PD in this study was in the mild to moderate stages of disease severity [Hoehn and Yahr (H&Y) = 2.3]. Previous research demonstrated that compared to HOAs, persons with PD who were in the later stages of the disease (H&Y = 4 and 5) have greater impairment in inhibitory gating (Teo et al., 1997). Due to greater degeneration in various parts of the brain (e.g., locus coeruleus, prefrontal cortex, basal ganglia, etc.) with the progression of PD, stress may have a differential impact on inhibitory gating. Further research is needed to determine the impact of disease progression on inhibitory gating in PD.

### Bradykinesia and tremor

As expected, compared with HOAs, persons with PD had a smaller movement amplitude for finger tapping and the pronation-supination of the hand. Persons with PD also demonstrated slower pronation-supination of the hand. Finally, persons with PD had more postural tremor and resting tremor. These results suggest that the quantitative analysis used was sensitive enough to capture PD motor symptoms of bradykinesia and tremor.

The results revealed that the SECP task decreased finger tapping amplitude in both groups, with a larger (non-significant) difference in persons with PD (*MD* = −0.54 cm) than HOAs (*MD* = −0.23 cm). Moreover, the SECP task increased finger tapping amplitude variability in persons with PD, while the amplitude variability in HOAs decreased. Previous research has shown that impairments in upper limb repetitive movements in PD are rate-dependent, with greater impairment at movement rates above 2 Hz (Freeman et al., 1993; Nakamura et al., 1978; Stegemöller et al., 2009; Yahalom et al., 2004). However, movement rate did not change for finger and hand movements during the SECP task, which suggests that any changes in amplitude mediated by the SECP task may not be explained by changes in movement rate. Furthermore, the SECP task increased amplitude variability in persons with PD and decreased amplitude variability in HOAs. This suggests that while the SECP task may decrease amplitude in both persons with PD and HOA, the underlying mechanisms may be different. Further research to delineate the differences in finger tapping performance is needed. Nonetheless, given that finger tapping performance is significantly related to the performance of more complex tasks, such as Purdue Pegboard (Müller et al., 2000), buttoning (Uzochukwu and Stegemöller, 2019), handwriting (Stegemöller et al., 2019), and postural instability (Stegemöller et al., 2016) in persons with PD, stress may further exacerbate more complex movements. Indeed, previous research has found that stress negatively impacted skilled reaching in a PD rat model (Smith et al., 2008) and that a stressful environment negatively impacted gait in persons with PD (Ehgoetz Martens et al., 2015). Yet, the SECP did not significantly impact hand movements or pronation-supination of the hand in this study, possibly due to the small sample size. Continued research to better understand the impact of stress on motor impairment is needed.

Postural tremor in both persons with PD and HOAs increased after the SECP task, with persons with PD showing a larger increase in postural tremor than HOAs. Increases in tremor were not completely unexpected, as others have shown that stress can increase postural tremor in healthy young adults (Mitchem and Tuttle, 1954). Specifically, increasing catecholamines associated with stress, such as norepinephrine, epinephrine, and acetylcholine, can increase postural tremor in healthy young adults (Marshall and Schnieden, 1966). However, resting tremor, a cardinal symptom of PD, did not significantly change with the SECP task. Evidence suggests that resting tremor may have a different underlying pathophysiology than postural tremor (Louis et al., 1999; Louis et al., 2001). Resting tremor has been shown to be mediated by the loss of dopaminergic neurons in the retrorubral area (Hirsch et al., 1992; Jellinger, 1999; Oiwa et al., 2003). While evidence is sparse, stress does not appear to impact dopaminergic output of the retrorubral area (Deutch and Roth, 1991). Thus, stress induced by the SECP task may have increased postural tremor by releasing catecholamines, while the networks and neurotransmitters that mediate resting tremor were unaffected. Future research confirming this notion is needed.

### Relation between inhibitory gating and motor impairment

Sensory processing impairments are hypothesized to contribute to motor impairments in persons with PD (Conte et al., 2013; Konczak et al., 2009; Müller et al., 2013; Nieuwboer and Giladi, 2013). Persons with PD demonstrate impairments in inhibitory gating in early and mid-latency components (Gulberti et al., 2015; Lukhanina et al., 2009; Lukhanina et al., 2011; Teo et al., 1997). Inhibitory gating has been associated with bradykinesia symptoms but no other MDS-UPDRS motor scores (Lukhanina et al., 2011). The results of this study are in line with the previous research. However, the study by Lukhanina et al. (2011) used the subjective ordinal MDS-UPDRS rating scale, which is unable to detect small differences in specific kinematic components, such as amplitude and frequency. Thus, the results of this study add to the literature suggesting that inhibitory gating may be related to movement amplitude impairment in some motor symptoms of PD.

While the exact mechanisms of the negative impact of stress on PD inhibitory gating and motor symptoms are unknown, there are three potential pathways that could explain the results observed in this study. First, decreases in inhibitory gating may result in sensory overloading, which may impair cognitive functioning by competing for limited cognitive resources (Croft et al., 2001; Desimone and Duncan, 1995; Yadon et al., 2009). In turn, this could affect movement by negatively impacting the ability of persons with PD to use cognitive mechanisms to compensate for basal ganglia damage and the resulting movement impairments. Second, stress may also negatively affect PD motor symptoms by disrupting normal sensory processing. Finally, PD motor symptoms may be negatively impacting other inhibitory processes that are associated with inhibitory gating, such as motor inhibition (Cheng et al., 2016; Liu et al., 2011; Yadon et al., 2009), which is also impaired in persons with PD (Obeso et al., 2011). In short, there are a number of possible mechanisms by which inhibitory gating may influence PD motor symptoms. Additional research is warranted to examine these relationships.

Finally, variability in participant characteristics may have contributed to the findings. Participants with PD have higher trait anxiety scores than the HOAs in this study. Higher anxiety alone may have independently modulated both inhibitory gating and motor impairment. Participants with PD in this study were all asked to adhere to the same timing of medication administration on the study day, but the dosage and effects of the dosage likely vary from participant to participant. These differences in medication may also have the potential to modulate both inhibitory gating and motor impairment. Consideration of these additional factors is important in the interpretation of the results. Future studies should consider including larger sample sizes that will allow for adequate control and analysis of participant characteristics, such as anxiety and medication.

### Limitations

While our sample size was large enough to support our initial hypothesis that the task reduced inhibitory gating in both HOAs and persons with PD, we were likely underpowered to find a group by condition interaction. However, the effect sizes support the notion that stress may reduce inhibitory gating in individuals with PD to a greater degree. Moreover, significant associations between inhibitory gating and some motor impairments were revealed, contributing additional information to the literature. However, the sample size was small, increasing the risk of Type 1/11 error. Another limitation is that the SECP task included both a physical stressor (cold water) and a social evaluation. Thus, it is not possible to determine if the differences observed in this study were from nociceptive stress, social-evaluative stress, or their interaction. We also did not measure the effect of stress on more complex motor skills, such as walking, handwriting, or speaking. Whether stress negatively impacts complex motor skills in persons with PD remains to be examined. Moreover, not all cardinal symptoms, rigidity, gait, and postural instability were measured, which further limits the interpretation. However, given that bradykinesia and tremor impact functional motor skills, stress may still be considered an important modifiable factor. Finally, the potential mechanisms mediating neurotransmitters, motor symptoms, and inhibitory gating were not assessed, and future studies are needed to clarify how stress might impact these in persons with PD.

## Conclusion

The SECP task decreased inhibitory gating, decreased finger tapping amplitude, and increased postural tremor in both persons with PD and HOAs, with these effects being larger in persons with PD. Moreover, the decreased inhibitory gating was related to changes in the movement amplitude of finger tapping and postural tremor. Although the sample size was small and there are limitations, this study adds to the limited research aimed at understanding the impact of stress on motor symptoms in PD. Understanding the impact of stress and inhibitory gating in persons with PD has the potential to further unlock important underlying mechanisms and neuropharmacological targets for treatment.

## Data Availability

The raw data supporting the conclusions of this article will be made available by the authors, without undue reservation.

## Footnotes

^1^ https://www.krigolsonlab.com/data-analysis.html
